# Gibberellin Increases the Bud Yield and Theanine Accumulation in *Camellia sinensis* (L.) Kuntze

**DOI:** 10.3390/molecules26113290

**Published:** 2021-05-29

**Authors:** Wei Li, Fen Xiang, Yi Su, Zhoufei Luo, Weigui Luo, Lingyun Zhou, Hongyan Liu, Langtao Xiao

**Affiliations:** 1 Hunan Provincial Key Laboratory of Phytohormones and Growth Development, Hunan Agricultural University, Changsha 410125, China; tea702@163.com (W.L.); yisu@hunau.edu.cn (Y.S.); zhoufeiluo@hunau.edu.cn (Z.L.); wg1122@126.com (W.L.); 2 Tea Research Institute, Hunan Academy of Agricultural Science, Changsha 410125, China; xiangfen-1210@163.com (F.X.); zhoulingyun0808@126.com (L.Z.); liuhongyan1218@126.com (H.L.)

**Keywords:** *Camellia sinensis*, theanine, gibberellin, chlorophyll fluorescence

## Abstract

Tea (*Camellia sinensis*) is one of the most important cash crops in the world. Theanine, as an important amino acid component in tea, is a key quality index for excellent tea quality and high economic value. People increase theanine accumulation in tea mainly through the application of nitrogen fertilizer, shading and pruning. However, these methods are not effective. In this study, we treated tea buds with a 100 μM solution of GA_3_ containing 1‰ tween-20, investigated the effects of GA_3_ on theanine accumulation, bud yield, chlorophyll fluorescence parameters and expression level of theanine biosynthesis pathway genes in tea plant by qPCR, LC-MS/MS etc. Results showed that change trends of theanine and GA_3_ was extremely positively correlated with each other. Exogenous GA_3_ upregulated the expression level of theanine biosynthesis pathway genes, caused an increase of theanine content (mg·g^-1^) by 27% in tea leaves compared with Mock, and accelerated the germination of buds and elongation of shoots, which lead to a significant increase of tea yield by 56% (*w*/*w*). Moreover, the decrease of chlorophyll contents, photochemical quenching coefficient (*qP*) and relative electron transport rate (*rETR*) under GA_3_ treatment suggested that GA_3_ reduced photosynthesis in the tender tea leaves, indicating that the decline of carbon assimilation in tea plants was conducive to the nitrogen metabolism, and it was beneficial to the accumulation of theanine. This study provided a new technical and theoretical support for the precise control of tea quality components and phenophase.

## 1. Introduction

Humans have consumed tea (*Camellia sinensis* (L.) Kuntze) as daily necessities for thousands of years, because of the health-promoting functions [1,2,3,4]. In market, the consuming tea categories were mainly composed of the green tea, black tea, white tea, dark tea, yellow tea and oolong tea according to the tea processing method. Among them, the consumption of green tea is the second largest tea category after black tea that reaches 8.8 × 10^5^ tons per year (http://www.statista.com, accessed on 19 April 2021) [5,6]. Compared with other tea categories, green tea is rich in theanine, thus the content of theanine in green tea has gained more concern. Through a long-term domestication and selection in different ecological environments, many tea cultivars were developed for making green tea, such as Longjing 43, Yabukita, Yatakamidori, Ngoc Thuy and Baojinghuangjincha 1# (HJ1) [2,7,8]. Among them, HJ1, with higher NUE, higher levels of amino acids and higher leaf yield compared with Fudingdabaicha (a common tea cultivar used as control), is native to a relatively isolated natural environment in the Wuling mountains of Western Hunan, China [2]. Although a certain tea cultivar shows a specific taste caused by multiple components, it is generally acknowledged that the type and contents of free amino acids are the key factors contributing to tea taste to a large extent and even determine tea quality and economic value [9]. l-theanine (γ-glutamyl-l-ethylamide), a peculiar amino acid in tea, is drawing more attention in the areas of tea production and research. Generally, theanine accounts for over 50% of total free amino acids in the dry tea products [10]. As a specific free amino acid, theanine shows good absorption and transportation characteristics in human body, and thus it is allowed to cross the blood–brain barrier and has multiple health-promoting roles, such as anti-anxiety, anti-tumor, neuron-protecting and memory improving [11]. Therefore, theanine has gained increasing attention in the modern tea cultivation and related fundamental research recently [10,12].

Theanine, as a natural ethylamide analogue of glutamate [13,14], is synthesized in vivo from glutamate and ethylamine (EA) by theanine synthetase (TS, EC 6.3.1.6) mainly in the root. In addition, other enzymes in plant, such as Gln synthetase (GS, EC 6.3.1.2), can also catalyze glutamate and EA to produce theanine [15]. Theanine also can be transported to the shoots and regionalized in the vacuole of mesophyll cell as a major nitrogen reservoir [10]. The accumulation of theanine in leaves was closely related to the leaf maturity grade in tea plant [16,17,18]. Comparing to the old leaves and stems, the young leaves contain a higher level of theanine. Theanine synthesis in tea plants is involved in a series of processes controlled by a variety of internal and external factors including the genotypic background, climate and cultivation conditions. Recently, some genes coding theanine biosynthesis enzymes were identified in tea plants through the genome sequencing, such as *CsTS1* and its homologous genes *CsGSII* [10,15,19]. The theanine accumulation is directly affected not only by the natural environment, but also by the agronomic measures, e.g., nitrogen supply, shading rate and plant growth regulators. These factors could significantly affect the expression level of *CsTS1* and *CsGSII* [20,21,22].

Gibberellins (GAs) are a class of important phytohormones synthesized in plant cell, and have made significant contributions to the “green revolution” in agricultural industry [23,24]. In plants, GAs play very important roles in lateral bud germination, stem elongation and flower development [25,26,27]. GAs and its biosynthesis inhibitors (e.g., paclobutrazol, uniconazole) are usually applied in controlling the rice heading, apple scion height and pea stem elongation, etc. [28,29,30,31]. These compounds are also used to regulate the biosynthesis and accumulation of soluble sugar in barley and glutamine in pine [32,33,34,35]. Intriguingly, GAs also emerge as important regulating factors in the biosynthesis and accumulation of various amino acids such as glutamine and gamma-aminobutyric acid [32,34,36]. Moreover, gibberellin content in tea increased first and then decreased during the stages from overwintering buds to mature leaves in spring, which seems to be consistent with the change trend of main free amino acid, such as theanine, indicating that gibberellin may participate in the regulation of theanine metabolism [27,37,38]. Meanwhile, theanine metabolism occurring at the germination stage is a source of energy and nutrients for new tissues [39], and this process is probably also controlled by GAs through its signaling pathway-related transcription factors such as CsMYB12 and CsMYB94 [12,40,41]. However, there are few reports about gibberellin directly affecting theanine accumulation. In the past few decades, GAs as plant growth regulators have been widely applied in agricultural production, but rarely employed in the cultivation of tea, although GAs showed a great application potential in amino acids and quality improvement.

In order to clarify the effects of gibberellins on tea yield and quality, GA_3_ and uniconazole (UZ) were employed to treat tea plants in this study. We focused on the dynamic synthesis of theanine at the stages of bud germination, to explore if GA_3_ has a promoting function on the theanine accumulation and quality in tea and its physiological and molecular mechanisms, and aimed to provide a new way to significantly increase the quality of tea.

## 2. Results

### 2.1. Dynamic Accumulation of Gibberellin and Theanine During Tea Bud Germination and Elongation

**Figure 1:**
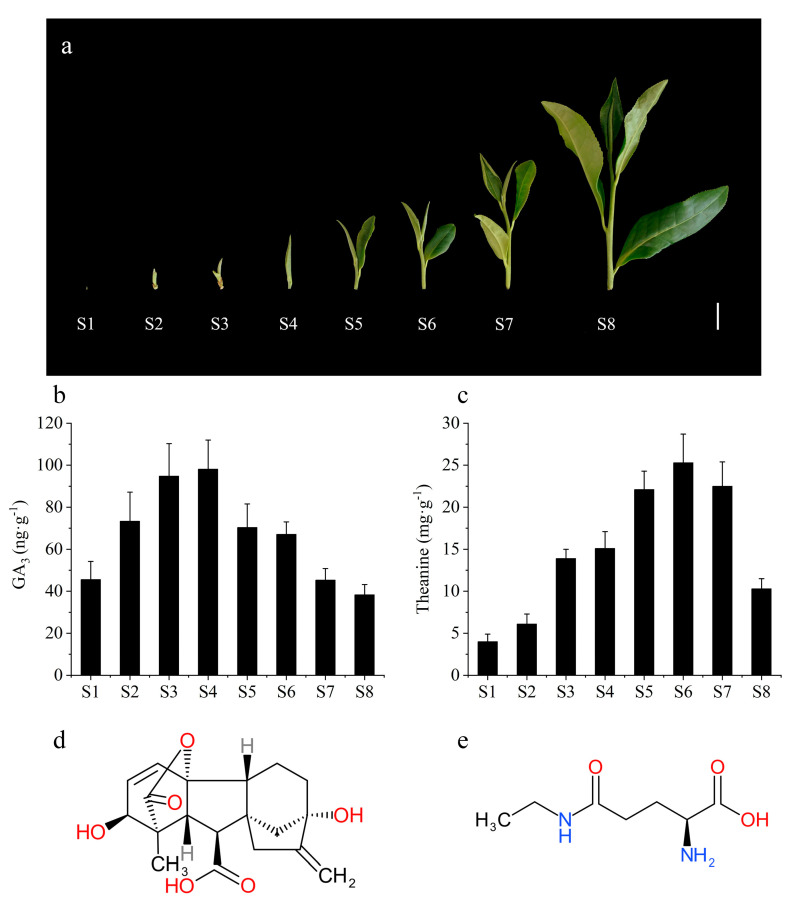
The morphological character of tea (*Camellia sinensis*) buds (**a**), and accumulations of GA_3_ (**b**) and theanine (**c**) in tea buds at different growth stages. (**d**,**e**) represent the chemical structure of GA_3_ and theanine. S1–S8 represent the stage of overwintering bud, bud germination, bud elongating, one bud, one bud with one leaf, one bud with two leaves, one bud with three leaves and one bud with four leaves, respectively. The values represented the mean ± SE of 10 biological replicates (n = 3) in (**b**,**c**).

According to the growth status and the tea plucking standard, the tea shoots are classified into eight stages (Figure 1a). The content of total free amino acid showed significant differences at different bud stages. In general, the tea at the one bud with one leaf or two leaves stage (S5–S6) tastes better and thus is more popular for people, because the contents of total free amino acids (including theanine) are relatively higher at S5–S6 stage. In fact, the bud development and metabolites are tightly controlled by multiple cellular regulators. Among them, GAs played a key role in bud germination and stem elongation in plants. In the previous study, we found that the gibberellin signal was closely related to the accumulation of theanine [2]. In order to reveal the dynamic correlation between the GAs and theanine, the contents of theanine and GAs (GA_1_, GA_3_, GA_4_, GA_7_) were monitored at different bud stages in this study. The results showed that GA_3_ sharply increased at the bud germination (S2) and elongation (S3) stages, and achieved peak level at the one bud (S4) stage (Figure 1a,b). At the following stages, GA_3_ content rapidly decreased to the previous level (Figure 1a,b). The dynamic changing trend of theanine content was similar with GA_3_ level, although the peak of theanine content was achieved at the S6 stage, which was later than GA_3_ (Figure 1b,c). The complex signal transduction mediated by gibberellin may result in the late theanine synthesis and accumulation comparing to the GA_3_ changes. Therefore, we analyzed the correlation between GA_3_ (S1–S8) and theanine two periods in advance (S3–S10). The results showed that there was a significant positive correlation between them (Table S1), indicating that GA_3_ is an important positive regulator mediating theanine accumulation. 

### 2.2. Gibberellin Treatment Promotes the Lateral Bud Germination and Elongation

**Figure 2:**
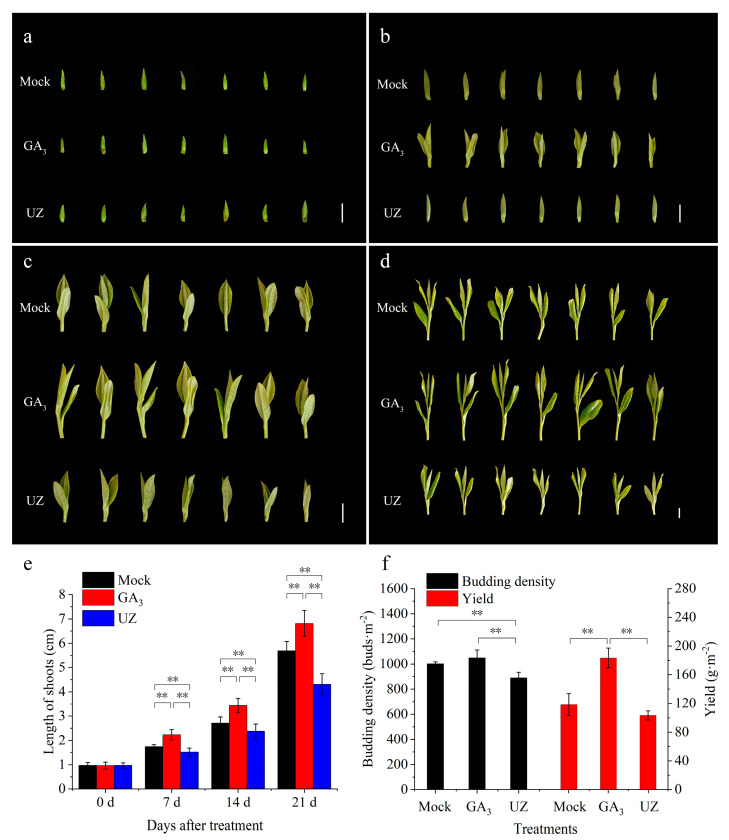
Effects of exogenous GA_3_ and gibberellin biosynthesis inhibitor (UZ) on bud germination, growth and yield of *Camellia sinensis*. The phenotypes of tea buds treated with Mock, GA_3_ and UZ at 0 (**a**), 7 (**b**), 14 (**c**) and 21 (**d**) days after spray treatment; length of tea shoots after GA_3_, Mock and UZ treatments at 0 d, 7 d, 14 d and 21 d (**e**), and theanine content in tea shoots after GA_3_, Mock and UZ treatments at 0 d, 7 d, 14 d and 21 d (**f**). The bar in (**a**–**d**) represents one centimeter. The values represented the mean ± SE of 30 and 6 biological replicates in (**e**,**f**) respectively (n = 3, Duncan’s multiple range test). **: Extremely significant difference (*p* < 0.01).

We treated the tea buds both with GA_3_ and gibberellin biosynthesis inhibitor (UZ) to ascertain the effects of gibberellin on the growth and development of tea shoots (Figure 2a). At 7 d after treatment, the first leaf outgrew under GA_3_ treatment, while the buds in the other groups remained in single bud shape (Figure 2b). At 14 d, the buds treated with GA_3_ grew to one bud and two leaves, whereas the tea plant in Mock and UZ group remained with one bud and one leaf or one bud and two leaves in early development (Figure 2c). At 21 d, the buds treated with GA_3_ grew to one bud and three leaves, while the buds in other two treated tea plants were still at the stage of one bud and two leaves (Figure 2d). The length of tea shoots treated with GA_3_ was significantly higher than the Mock at 7, 14 and 21 days (Figure 2e). At same time, after the UZ treatment, we found that the shoots were significantly shorter compared to Mock (Figure 2e). The results showed that GA_3_ promoted the bud germination and shoot growth (Figure 2b–d), and thus a certain effect to improve tea yield.

As an important cash crops, the value of tea is closely related to its yield and quality. To understand the effects of bio-stimulators on yield of tea buds, budding density and tea yield were detected at 14 d after treatment. The results showed that GA_3_ caused the increase of tea budding density comparing to Mock (Figure 2f). Due to the promotion effect of GA_3_ on shoot growth (Figure 2c), the yield treated with GA_3_ was increased remarkably by 56% (*w*/*w*) compared with Mock (Figure 2f). On the contrary, UZ significantly inhibited the germination and growth of tea buds, resulting in a significant reduction of 12% (W/W) in tea yield.

### 2.3. Gibberellin Treatment Increased the Theanine Content in Tea Buds and Leaves

**Figure 3:**
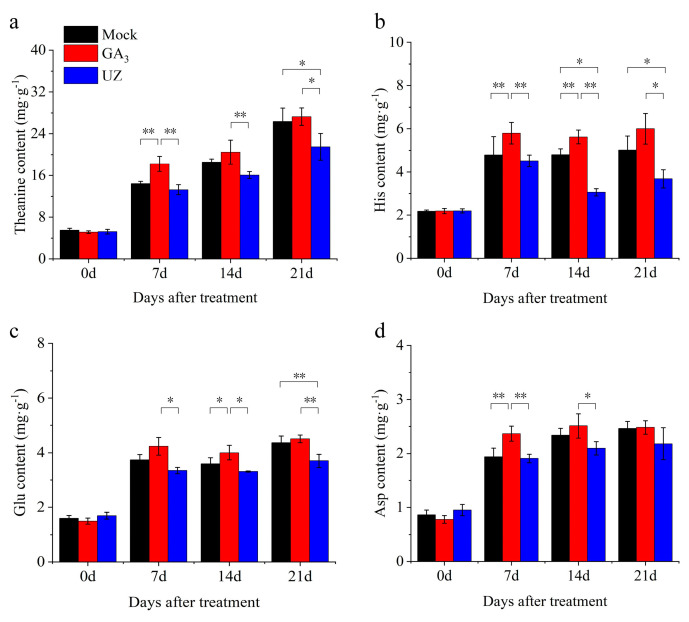
Response of several abundant amino acids to GA_3_ in tea leaves. (**a**)–(**d**) The change of theanine, histidine (His), glutamate (Glu) and aspartate (Asp) content respectively at 0–21 d after treatment. The values represented the mean ± SE of 30 biological replicates (n = 3, Duncan’s multiple range test). *: Significant difference (*p* < 0.05); **: Extremely significant difference (*p* < 0.01).

The theanine content in tea buds is one of the decisive factors of tea quality, which is tightly associated with the bud growth. In this study, we detected the amino acid compositions during the process of the bud germination and growth after treatments. As the main component in free amino acids, theanine showed an increasing trend after each treatment, and theanine content under GA_3_ treatment was significantly higher by 27% than that of Mock at 7d (Figure 3a, Table S3). In addition, the content of theanine in tea treated with GA_3_ did not decrease after S6 (Figure 3a), indicating that GA_3_ has a positive role in the maintenance of high level of theanine in tea. Compared with UZ, the differences between them were significant at 7d, 14d and 21d. The content of theanine after UZ treatment was lower than that of Mock, while there was significant difference between UZ and Mock at 21d after treatment, which indicated that GA_3_ has a rapid effect on the theanine accumulation (Figure 3a). The lower GA_3_ in tea plant treated with UZ resulted in the slower response of theanine content to UZ. Along with the continuous growth of buds, the differences of theanine content in buds were gradually decreased between GA_3_ and Mock at 14 d after treatments (Figure 3a). In addition, GA_3_ not only increased theanine content, but also increased the content of several other amino acids, such as His, Glu and Asp (Table S3). The trends in contents of the above free amino acids (accounting for more than 5% of the total free amino acids respectively) in tea was basically consistent with theanine (Figure 3b–d, Table S3).

### 2.4. Effects of GA_3_ on Gene Expression in Biosynthesis of Theanine in Tea Plants

**Figure 4:**
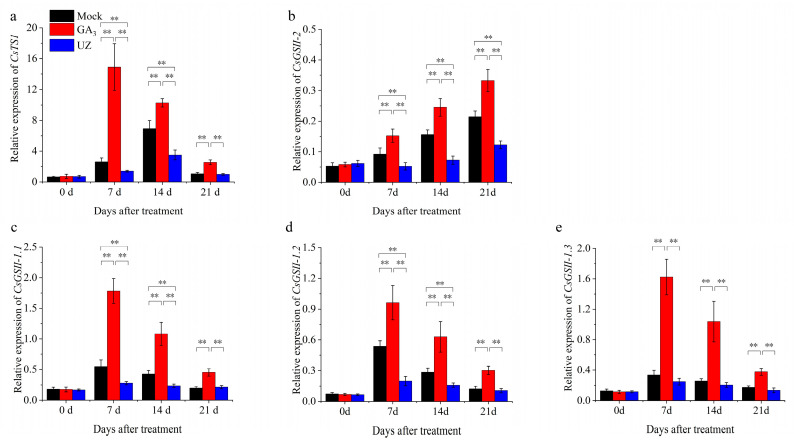
Relative expression of genes in theanine biosynthesis pathway in tea shoots. (**a**)–(**e**) The relative expression of *CsTS1*, *CsGSII-2*, *CsGSII-1.1*, *CsGSII-1.2* and *CsGSII-1.3* in tea shoots at 0–21d after treatment. The values represented the mean ± SE of 10 biological replicates (n = 3, Duncan’s multiple range test). **: Extremely significant difference (*p* < 0.01).

To understand the molecular mechanism of GA_3_ enhancing the content of theanine in tea plants, we further analyzed the expression levels of *CsTS1*, *CsGSII-1.1*, *CsGSII-1.2*, *CsGSII-1.3* and *CsGSII-2*, which were identified as the theanine biosynthesis pathway genes in the buds and leaves. As shown in Figure 4, *CsTS1*, *CsGSII-1.1*, *CsGSII-1.2*, *CsGSII-1.3* remarkably increased and achieved peak in shoots treated with GA_3_ at 7 d (Figure 4a, c, d, e). Then their relative expression decreased gradually. However, the relative expression of *CsTS1*, *CsGSII-1.1*, *CsGSII-1.2*, *CsGSII-1.3* were still significantly higher than that of Mock 21 d after GA_3_ treatment. Therefore, we can infer that the positive regulation of GA_3_ on these genes promotes the rapid synthesis and accumulation of theanine, and the difference among them tends to be flat with the degradation of GA_3_ and the feedback regulation of nitrogen metabolism in tea plants. The relative expression of *CsGSII-2* was significantly increased after GA_3_ treatment, and its expression level increased gradually (Figure 4b), indicating that GA_3_ is beneficial to increase the rate of nitrogen assimilation in this metabolic pathway. On the contrary, UZ inhibits the expression level of *CsTS1*, *CsGSII-1.1*, *CsGSII-1.2*, *CsGSII-1.3* and *CsGSII-2*, which is not conducive to the accumulation of theanine and the enhancement of nitrogen metabolism.

### 2.5. Gibberellin Affect the Chlorophyll Fluorescence Parameters in Tea Leaves

**Figure 5:**
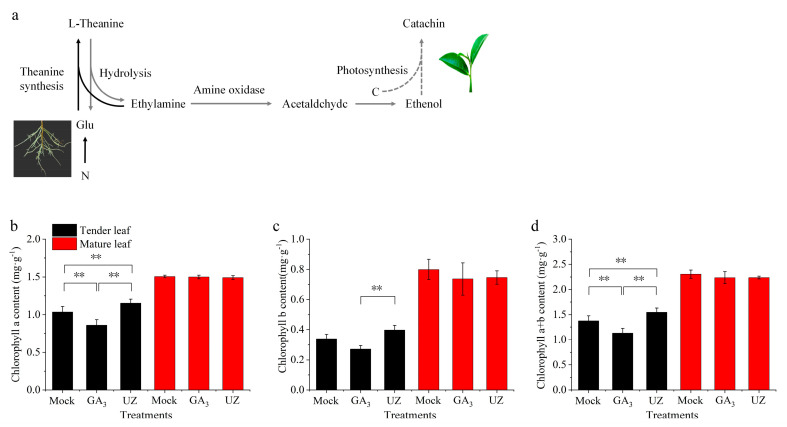
Chlorophyll response to GA_3_ and gibberellin biosynthesis inhibitor (UZ) treatments in leaves of *Camellia sinensis*. (**a**) The pathway between theanine metabolysis and catechin biosynthesis. Black arrows represent the pathway of theanine biosynthesis, gray arrows indicate the pathway that may be involved in catechin biosynthesis after theanine hydrolysis. (**b**)–(**d**) The chlorophyll a, chlorophyll b and chlorophyll a + b contents, respectively, in tender leaf (second leaf from top of the shoot) and mature leaf. The values represented the mean ± SE of 4 biological replicates (n = 3, Duncan’s multiple range test) in (**b**)–(**d**). **: Extremely significant difference (*p* < 0.01).

As the most abundant free amino acid in tea, theanine is mainly formed in the root and transported to the tea shoots by the vascular system. Theanine is also a main intermediate storage nitrogen source for nitrogen metabolism, and is closely related to carbon metabolism of tea plants [13,42] (Figure 5a). The metabolites of theanine partly participate in the biosynthesis of catechin in tea leaves (Figure 5a) [17]. In this study, we determined the chlorophyll content and chlorophyll fluorescence parameters which are closely related to the carbon metabolism of tea plants. The chlorophyll a, b and chlorophyll a + b content were reduced in tender leaves after GA_3_ treatment (Figure 5b–d). By contrast, compared with Mock, chlorophyll a, b and chlorophyll a + b content were remarkably increased in tender leaves after UZ treatment. There was no significant difference in chlorophyll content in mature leaves after GA_3_ and UZ treatments.

**Figure 6:**
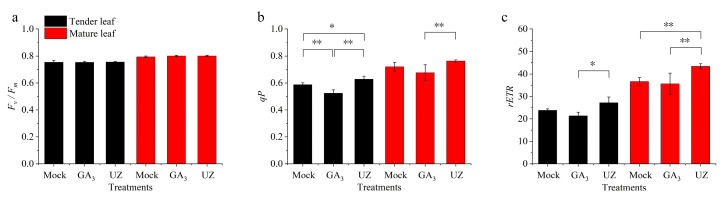
Effect of GA_3_ and gibberellin biosynthesis inhibitor (UZ) on the leaf chlorophyll fluorescence parameters of *Camellia sinensis*. The chlorophyll fluorescence *F_v_*/*F_m_* (**a**), *qP* (**b**), and *rETR* (**c**) in tender leaf and mature leaf. The values represented in (**a**)–(**c**) is the mean ± SE of 4 biological replicates (n = 4, Duncan’s multiple range test). *: Significant difference (*p* < 0.05); **: Extremely significant difference (*p* < 0.01).

The variation of leaf chlorophyll senescence parameters showed that GA_3_ and UZ did not change the maximal photochemical efficiency (*F_v_*/*F_m_*) in tender leaves (Figure 6a), but the photochemical quenching coefficient (*qP*) and relative electron transport rate (*rETR*) was declined significantly after GA_3_ treatment (Figure 6b,c), which was consistent with the variation of chlorophyll, suggesting that GA_3_ reduced photosynthesis of tender leaves of tea plant. On the contrary, the *qP* and *rETR* were raised by gibberellin biosynthesis inhibitor (UZ) compared with Mock and GA_3_. The variation of chlorophyll senescence parameters in mature leaves were generally consistent with tender leaves (Figure 6a–c).

We further analyzed the correlation between theanine content, chlorophyll a+b content and *qP* in tender leaf of tea shoot among different treatments. The results showed that theanine was negatively correlated with chlorophyll a+b and *qP* (Table 1), indicating that the decrease of photosynthetic efficiency is beneficial to theanine accumulation and tea quality improvement.

## 3. Discussion

The content of free amino acids in tea is one of the most important indexes for tea quality, and the ratio of polyphenols to amino acids in tea determines the taste. As the major component of amino acids in tea leaves (about 50%), theanine decides the umami and sweet taste of tea, thus it is critical for the quality of tea. In tea production, theanine in tea plant is usually regulated through agronomic measures, such as fertilization, pruning, shading, etc. However, these methods are time-consuming and less effective. Therefore, it is necessary to develop a rapid and effective regulation measure for theanine accumulation.

The dynamic change of theanine accumulation is closely related to gibberellins. Theanine increased rapidly along with the germination and growth in tea buds [42], and it is a functional component with important biological activities and broad development at prospects during the buds break and growth in tea shoots. Recent research has shown that changes in amino acids profiles were associated with the release of buds from dormancy [43,44]. For phytohormones, gibberellins play important roles not only on the bud break and growth, but also on amino acid metabolisms [32,33,34,35]. In this study, the effects of gibberellins on theanine accumulation were investigated, and the change trends of GA_3_ and theanine content were very similar to each other, indicating the tight correlation between gibberellins and theanine (Figure 1). Moreover, gibberellins have a certain lag in regulating theanine synthesis through its signaling pathway (Figure 1, Table S1). Combining with the theanine accumulation and germination of tea buds treated with GA_3_ and UZ, we infer that exogenous GA_3_ application exerts a strong positive regulation on theanine accumulation (Figure 2). We also analyzed the effects of GA_3_ and UZ on the expression of theanine biosynthesis pathway genes (*CsTS1*, *CsGSII-2*, *CsGSII-1.1*, *CsGSII-1.2* and *CsGSII-1.3*) (Figure 4), and the results confirm the inference above.

GA_3_ promotes theanine accumulation by reducing the photosynthetic efficiency of tea shoots. Theanine accumulation in tea leaves may be closely related to photosynthetic efficiency [21,45]. Chlorophyll content and *qP* are important parameters reflecting the light utilization efficiency of plant leaves. Due to the rapid growth and elongation of tea shoots after GA_3_ treatment, the chlorophyll content decreased relatively compared with Mock and UZ (Figure 5b–d). Further research showed that the differences of *F_v_*/*F_m_* among Mock, GA_3_ and UZ were not significant, neither in tender leaves nor mature leaves (Figure 6). Previous studies reported that moderate shading could improve the content of amino acids and decreased the catechin content in fresh leaves by carbon and nitrogen cycles regulation [21,45]. Our work also showed that GA_3_ treatment reduced the photochemical quenching (*qP*) and relative electron transport rate (*rETR*) both in tender leaves and mature leaves (Figure 6b,c), which supported that the decline of carbon assimilation in tea plants was conducive to the nitrogen assimilation, and it was beneficial to the synthesis and accumulation of the intermediate products such as theanine and other nitrogen metabolites (Figure 3, Table S3) [22]. On the contrary, UZ enhanced the *qP* and *rETR* (Figure 6b,c), which promoted photosynthetic efficiency and accelerate the decomposition of secondary metabolites such as theanine. Its hydrolyzate ethylamine was further oxidized to acetaldehyde by amine oxidase (AO) and was led to participate in catechin biosynthesis, which was unfavorable for the accumulation of theanine [17,21].

The economic value of tea is not only related to its quality and yield, but also restricted by the plucking time. The earlier the tea goes on the market, the higher its economic value [46]. Theanine content usually reaches the peak at S5–S6 stage and then sharply decreases (Figure 1), which leads to the decline of tea quality and taste, and further reduce its economic value. In this study, the growth stage of new shoots to S5 was 6–7 days earlier than that of mock after GA_3_ treatment (Figure 2a–d), thus not only increased the economic value of tea by the advance of plucking time, but also increased the yield of tea in the same grade (Figure 2f), and further improved the economic value.

## 4. Materials and Methods

### 4.1. Chemicals

GA_3_, Tween-20, *N*-(3-dimethylaminopropyl)-*N*0-ethylcarbodiimide (EDC) were purchased from Sigma-Aldrich (St. Louis, MO, USA). Standard compound theanine, uniconazole, were purchased from Solarbio Science & Technology (Beijing, China). Phosphoric acid (Hromatographic grade), methanol (MeOH), formic acid (FA), acetone, ethanol (EtOH) and ethyl acetate were purchased from China National Pharmaceutical Group (Shanghai, China). Acetonitrile (Hromatographic grade) was purchased from TEDIA Company (Fairfield, OH, USA).

### 4.2. Plant Materials and Treatments

The variety “Baojinghuangjin tea 1#” were planted in experimental tea plantation of Hunan Tea Research Institute and grown to tea industrial size over 10 years in Changsha, Hunan (113°4′31.37″ E, 28°12′23.49″ N). 

The tea plantation covers an area of 2,000 m^2^, with strip planting and a row width of 1.5 m. The tea garden was divided into 12 plots with an area of 150 m^2^ per plot. The plots were randomly distributed, and isolated rows were set among the plots. The plots were divided into 3 groups, each group contains 4 plots randomly. All of them were grown in the same condition and were pruned in November 2019.

The effects of different levels of GA_3_/UZ on the growth of tea shoots were studied (Table S4); three kinds of treatment solutions were made: 100 μM GA_3_ containing 0.1% tween-20 [47], 100 μM uniconazole containing 0.1% tween-20, and the Mock containing 0.1% tween-20.

As the overwintering buds (S1) of tea were not suitable for treatment and the buds (S4) were too late for treatment, the germinated buds about 1 cm long (S3) were treated with GA_3_, uniconazole and Mock solutions by using the spraying method.

### 4.3. Sampling

The buds were collected at the stages of overwintering bud (S1), bud germination (S2), bud elongating (S3), one bud (S4), one bud with one leaf (S5), one bud with two leaves (S6), one bud with three leaves (S7) and one bud with four leaves (S8). Each sample was divided into 2 parts. One was frozen with liquid nitrogen for gibberellin analysis, and the other was for theanine determination.

The treated shoots were respectively collected at 7 days, 14 days, and 21 days after treatment; samples collected before treatments were designated as Day 0 samples. The samples were divided into 3 parts, one for length measuring, one for the analysis of free amino acid component and other was frozen with liquid nitrogen for RNA extraction.

Twelve plots with 0.1 m^2^ per plot sampling areas were randomly selected from the canopy of tea plants, and sampling was repeated five times for each plot.

### 4.4. Free Amino Acids Determination

Free amino acids were extracted and detected as described by Wei [10]. Briefly, 0.2 g of freeze-dried tea shoots were ground into fine powder. Then 4.5 mL deionized water were added into the sample, which was incubated for 15 min in a water bath at 100 °C to extract free amino acids. After centrifugation at 6000 rpm for 10 min, the residues were re-extracted once as described above. The supernatants were merged and cooled to room temperature, and diluted with water to a volume of 10 mL. The merged supernatants were also filtered through a 0.22 μm membrane before HPLC analysis.

Free amino acids were detected using a e2695 Waters HPLC system (Waters, Milford, MA, USA) with the AccQ-Fluor Reagent Kit (Waters, catalog # WAT052880) according to the manufacturer’s specifications [6]. A Waters AccQ-Tag reversed-phase HPLC column (150 mm × 3.9 mm, 5 μm) was used at a flow rate of 1.0 mL·min^−1^. The column oven temperature was set to 37 °C. The detection wavelength was set to 248 nm for analysis. The mobile phase consisted of AccQ-Tag (1:10 *v/v*) (A) in water, chromatographic grade acetonitrile (6:10) (B) in water, and the 45 min of line-gradient elution was used to detect the free amino acids. Then, 10 μL of the filtrate was injected into the HPLC system for analysis.

### 4.5. RNA Extraction and Quantitative Real-Time PCR

Samples were ground into powder in a mortar using liquid nitrogen. Total RNA was extracted from 80–120 mg of powder using a TIANGEN RNAprep Pure kit (TIANGEN, catalog # DP441). An amount of 1–2 μg total RNAs were used for reverse transcription by Fast Quant RT kit (TIANGEN, catalog # KR106). As previously described, the cDNA was then diluted to 200 ng·μL^-1^ and was used for the qPCR with SuperReal PreMix Plus (TIANGEN, catalog # FP205) on the Bio-Rad CFX96 realtime system (Bio-Rad, Hercules, CA, USA) [2]. Three technical replicates were applied for the relative gene expression analysis. The gene-specific oligonucleotide primers used for the qRT-PCR are listed in Table S2. Reactions were performed at 95 °C for 15 min, 40 cycles of 95 °C for 10 s, and 60 °C for 32 s. *CsACTIN* was used as reference for qPCR data analysis. All qRT-PCRs were normalized using the cycle threshold (Ct) value corresponding to the reference gene.

### 4.6. Gibberellin Extraction and Quantification

Samples were ground into powder in liquid nitrogen, then the GAs was extracted from ground samples with 75% MeOH containing 5% formic acid for 12 h in dark at 4 °C, centrifugation was performed to remove solid impurities at 15,000 *g* for 10 min. Residue was extracted twice with 250 mL MeOH, and the supernatant was combined and concentrated in vacuum. Dried extract was dissolved with 200 μL ddH_2_O (pH 2.5) and extracted three times with 100 μL ethyl acetate, the upper organic phase was combined and concentrated in vacuum again. Afterwards, 50 μL EDC (20 mM in EtOH) was used in excess to push forward the derivatization reaction and incubate at 40 °C for 1 h. The solution was concentrated and redissolved with ddH_2_O, after centrifugation at 6000 rpm for 10 min, the supernatant was used for GAs detection [48].

UPLC−MS/MS Conditions: UPLC-MS/MS analysis was performed on a LCMS-8060 system (Shimadzu, Kyoto City Japan) referred to Li [49]. The prepared sample (10 μL) was injected into a reversed phase packed column (XR-ODS, 75 mm × 2.0 mm I.D., 1.6 μm, Shimadzu) installed for the LCMS-8060. It was eluted at column temperature of 40 °C and flow rate of 0.3 mL·min^−1^, with binary solvents of 0.1% FA in water (A) and 0.1% FA in ACN (B) at a programmed gradient from 99:1 A:B (*v/v*) to 50:50 A:B (*v/v*) over 31 min. The elution band was ionized by ESI online. The positive ions were detected by MS in MRM mode as follows: nebulizing gas flow 3 L·min^−1^, drying gas flow 10 L·min^−1^, heating gas flow 10 L·min^−1^, interface temperature 300 °C, desolvation lines temperature 300 °C, heat block temperature 400 °C, and the dissociation gas induced by collision is 230 kpa.

### 4.7. Chlorophyll Fluorescence Determination

Chlorophyll fluorescence kinetics parameters were measured with a portable chlorophyll fluorometer PAM 2500 (Walz, Effeltrich, Germany) [3]. The tender leaves (second leaf from top of the shoot) and mature leaves from annual shoots of each treatment group were randomly selected for Chlorophyll fluorescence test. Prior to measurements, these leaves were kept in dark for 30 min using leaf clips in order to ensure any chlorophyll fluorescence yield fully quenched. Fluorescence origin (*F_o_*) and maximum fluorescence yield (*F_m_*) were measured, and results expressed as the effective quantum yield of PSII and in terms of the ratio of variable to maximum chlorophyll fluorescence (*F_v_*/*F_m_* = (*F_m_*−*F_o_*)/*F_m_*).

### 4.8. Chlorophyll Determination

The tender leaf (second leaf from top of the shoot) and mature leaf tissue (~ 0.5 g) was ground to a fine powder in liquid nitrogen, total chlorophyll was extracted with 8 mL 80% cold acetone for 8 h at 4 °C, and shaken or each tube was inverted for 30 s every 2 h. Centrifugation was performed to remove solid impurities at 6000 g for 10 min, and supernatant was diluted to 10 mL with 80% acetone. The chlorophyll content in the extract was quantified spectrophotometrically (T600, Pgeneral, Beijing, China) by measuring its optical density at 663 and 645 nm.

## 5. Conclusions

In this study, we investigated the possible correlation between gibberellin and theanine and the effects of GA_3_ on theanine accumulation. The change trends between the dynamic accumulation of GA_3_ and theanine was very similar, showing a significantly positive correlation with a correlation coefficient of 0.85. The effect of GA_3_ on new shoot of tea plant was investigated, the results showed that GA_3_ significantly increased theanine content (mg·g^−1^) by 27% and increased tea yield by 56% (*w/w*). In addition, GA_3_ negatively affected the chlorophyll content in tender leaves of tea plants. The decrease of chlorophyll content caused a significant reduction of photosynthetic efficiency in tender leaves. In conclusion, GA_3_ showed an ameliorative effect on theanine accumulation as well as nitrogen metabolism in tea plants, possibly through the decrease of photosynthesis and carbon assimilation. This study explained the mechanism of gibberellin on theanine accumulation from the perspective of carbon and nitrogen cycle, provided a theoretical basis for the precise control of tea quality components and phenophase, and has a broad application prospect for improving the economic value of tea.

## Figures and Tables

**Table 1:** Correlations between theanine content, chlorophyll content and quenching coefficient in tender leaf of tea plant.

	Chlorophyll a + b Content	Quenching Coefficient	Theanine Content
Chlorophyll a + b content	1		
Quenching coefficient	0.999 *	1	
Theanine content	−0.980 *	−0.990 *	1

*: significant correlation (*p* < 0.05).

## Data Availability

All of the recorded data are available in all Tables and Figures in the manuscript.

## Supplementary Materials

The following are available online, Table S1: Correlation coefficient between gibberellin and theanine accumulation during lateral bud germination and elongation. Table S2: Primers used for qRT-PCR assays in tea leaves. Table S3: The change of amino acid compositions in 0–21 d after treatment. Table S4: Effects of different levels of GA3/UZ on the growth of tea shoots.

## Sample Availability

Samples of the compounds are available from the authors.
